# Suboptimal antidepressant use among inpatients and outpatients with symptoms of depression: a cross-sectional analysis of the POKAL core data set

**DOI:** 10.1007/s00406-025-01989-y

**Published:** 2025-03-12

**Authors:** V. Brisnik, M. Schechner, P. Landmesser, H. Schillok, P. Schoenweger, M. Rottenkolber, D. Lindemann, K. Lukaschek, C. Jung-Sievers, P. Falkai, P. Henningsen, G. Pitschel-Walz, H. Krcmar, A. Schneider, C. Haas, F. Gökce, J. Eder, L. Pfeiffer, V. von Schrottenberg, C. Teusen, M. Bühner, J. Gensichen, T. Dreischulte

**Affiliations:** 1 Institute of General Practice and Family Medicine, LMU University Hospital, LMU, Munich, Germany; 2 Graduate Program “POKAL, Predictors and Outcomes in Primary Care Depression Care” (DFG-GrK 2621), Munich, Germany; 3 Institute of Medical Data Processing, Biometrics and Epidemiology (IBE), Faculty of Medicine, LMU Munich, Munich, Germany; 4 Pettenkofer School of Public Health, LMU Munich, Munich, Germany; 5 Department of Psychiatry and Psychotherapy, LMU University Hospital, LMU Munich, Munich, Germany; 6 Department of Psychosomatic Medicine and Psychotherapy, University Hospital, Technical University of Munich, Munich, Germany; 7 School of Computation, Information and Technology, Technical University of Munich, Garching, Munich, Germany; 8 Institute of General Practice and Health Services Research, School of Medicine, Technical University of Munich, Munich, Germany; 9 Department of Psychology, Psychological Methods and Assessment, Ludwig Maximilian University of Munich, Munich, Germany

**Keywords:** Depression, Antidepressants, Primary care, Adverse drug reactions, Potentially inappropriate medication

## Abstract

We present a cross-sectional analysis of 1391 outpatients and 280 inpatients participating in subprojects of the Research Training Group POKAL, of whom 1609 had a PHQ-9 score ≥ 5 and 62 reported depression with antidepressant use. Antidepressant use was lower among outpatients than inpatients (28.5% vs. 82.5%), with higher levels of SSRI monotherapy (44.1% vs. 25.5%). Of antidepressant users, 80.1% had potentially inadequate treatment response, 21.7% high-risk use and of those with severe symptoms, 42.1% were potentially undertreated. Key risk factors were higher anxiety levels (for inadequate treatment response) and polypharmacy (for high-risk use), while previous depressive episode was protective against potential undertreatment.

## Introduction

Long-term conditions place a heavy burden on healthcare systems globally [1–3]. Many patients face multiple illnesses, and the overlap of mental and physical health conditions often results in fragmented, poorly coordinated care [4]. The Research Training Group on Predictors and Outcomes in Primary Depression Care (POKAL) focuses on improving diagnosis, management, and evidence-based care for patients with depression, particularly those with multimorbidity [5].

Antidepressants are crucial for managing severe depression but do not guarantee symptom relief [6]. Polypharmacy, defined as the use of five or more drugs [7], increases the risk of adverse drug reactions (ADRs) from drug-drug, drug-disease, and drug-age interactions [8]. Balancing antidepressant benefits and risks is therefore critical to optimize outcomes from antidepressant treatment.

This cross-sectional analysis of the POKAL core data set (POKAL-CDS) aims to identify opportunities for improving antidepressant use by examining potentially inadequate treatment response, potential high-risk prescribing, and potential undertreatment in inpatient and outpatient settings.

## Methods

### Data source

The data comprised a sample of 2741 patients aged 15 years or older, proficient in German, who participated in one of the 5 POKAL sub-studies and completed the POKAL-CDS between January 2022 and July 2024 in Southern Germany and Austria. Details of the POKAL studies are given in Table 1. Outpatients (n = 2438; 89.0%) were recruited from waiting rooms in general medical and psychotherapy practices and through social media, while inpatients (n = 303; 11.0%) were recruited from psychiatric hospital wards.

**Table 1:** Characteristics of POKAL sub-studies

Study name	Total N	Objective	Recruitment setting	Patients included in our study	
				Symptoms of depression	Antidepressant use
Study A (MiP3)	250^#^	To identify predictors of depression severity	University hospital ward	229 (91.6%)	194 (77.6%)
Study B (valdesy)	547*	To validate a new questionnaire for diagnosing depression	GP practices	214 (39.1%)	34 (6.2%)
Study C (psych-education)	112^#^	To adapt and test a depression psychoeducation program	GP practices	105 (93.8%)	30 (26.8%)
Study D (somatization study)	1311*	To explore associations of somatic symptom disorder with personality dysfunction and specific maladaptive traits	GP practices	606 (46.2%)	97 (7.4%)
Study E (SuprX)	521^#^	To validate a new questionnaire on suicidal ideation	GP practices, University hospital ward, online	517 (99.2%)	273 (52.4%)
Total	2741			1671	628

*The study sample included individuals with and without symptoms of depression

^#^The sample exclusively included individuals with symptoms of depression

### Data collection

The POKAL-CDS were collected via patient interviews using validated surveys or templates. Apart from socio-demographics, data was collected on (a) symptom severity of depression (via the Patient Health Questionnaire-9 (PHQ-9) [9]), (b) symptom severity of anxiety (via the Generalized Anxiety Disorder-7 survey (GAD-7) [10], (c) somatic symptom severity (via the Patient Health Questionnaire-15 (PHQ-15) [11]). Information on current medications was collected through patient interviews and, when available, cross-referenced with a standardized medication plan.

### Definitions and data analysis

Patients with symptoms of depression were defined as those with a PHQ-9 score ≥ 5 or a self-reported history of depression with current antidepressant use. Quantitative data are reported as medians with interquartile ranges (Q1-Q3), and categorical data as absolute and relative frequencies. Group differences were assessed using Chi-Square, Fisher’s exact, or Mann–Whitney U tests. All tests are two-sided and p-value ≤ 0.05 were considered statistically significant.

High-risk prescribing was identified using an expert-developed indicator set ([12], applying 12 of 37 indicators (32.4%) focusing on drug-drug interactions. Potential undertreatment was defined as no antidepressant use in moderately severe/severe symptoms of depression (PHQ-9 ≥ 15), and potentially inadequate treatment response as presence of moderate to severe symptoms (PHQ-9 ≥ 10) despite antidepressant use.

Logistic regression models examined risk factors for high-risk prescribing, potentially inadequate treatment response, and undertreatment. Variables from Table 2 and antidepressant use were analyzed using univariate and multivariate odds ratios (ORs), with final models selected through forward selection. Analyses were conducted using IBM SPSS Statistics, version 29.

**Table 2:** Characteristics of patients with symptoms of depression included in the study sample

	Total	Out-patient setting	In-patient setting	p-value (outpatient vs inpatient)
	No. of patients (%) (n = 1671)	No. of patients (%) (n = 1391)	No. of patients (%) (n = 280)	
Sociodemographic data				
Female (missing = 31)	1038 (63.3)	902 (64.9)	136 (54.2)	0.002
Median age (Q1-Q3) (missing = 30)	42 (29–55)	43 (31–56)	36 (25–50)	< 0.001
Age ≥ 65	154 (9.4)	152 (11.1)	2 (0.7)	
General education (missing = 61)				< 0.001
< 10 y of formal education	343 (21.3)	306 (22.8)	37 (13.9)	
10 y of formal education	426 (26.5)	376 (28.0)	50 (18.8)	
12–13 y of formal education	841 (52.2)	662 (49.3)	179 (67.3)	
Currently employed (missing = 35)	1071 (65.5)	932 (68.2)	139 (51.5)	< 0.001
Marital status (missing = 21)				< 0.001
Divorced/widowed/single	779 (47.2)	624 (45.2)	155 (57.8)	
Married or in a stable relationship	871 (52.8)	758 (54.8)	113 (42.2)	
Residence (missing = 112)				< 0.001
Urban area	1232 (79.0)	998 (77.3)	234 (87.3)	
Rural area	327 (21.0)	293 (22.7)	34 (12.7)	
Previous history of depression (missing = 22)	1004 (60.9)	730 (53.3)	274 (98.2)	< 0.001
Median no. of drugs taken (Q1-Q3) (missing = 0)	1 (0–3)	1 (0–3)	3 (2–4)	< 0.001
0–4 drugs	1466 (87.7)	1249 (89.8)	217 (77.5)	
≥ 5 drugs	205 (12.3)	142 (10.2)	63 (22.5)	
Median PHQ-9 score (Q1-Q3) (missing = 35)	11 (7–17)	10 (7–15)	18 (14.5–22)	< 0.001
No depressive symptoms (Score: 0–4)	27 (1.7)	22 (1.6)	5 (2.0)	
Mild symptoms (Score: 5–9)	610 (37.3)	593 (42.9)	17 (6.7)	
Moderate symptoms (Score: 10–14)	445 (27.2)	404 (29.2)	41 (16.2)	
Moderately severe/severe symptoms (Score: 15–27)	554 (33.9)	364 (26.3)	190 (75.1)	
Median GAD-7 score (Q1-Q3) (missing = 52)	9 (6–13)	8 (5–12)	13 (9–17)	< 0.001
Minimal symptoms of anxiety (Score: 0–4)	289 (17.9)	271 (20.0)	18 (6.8)	
Mild symptoms (Score: 5–9)	575 (35.5)	523 (38.7)	52 (19.5)	
Moderate symptoms (Score: 10–14)	443 (27.4)	355 (26.2)	88 (33.1)	
Severe symptoms (Score: 15–21)	312 (19.3)	204 (15.1)	108 (40.6)	
Median PHQ-15 score (Q1-Q3) (missing = 321)	12 (8–16)	11 (7–15)	17 (13–21)	< 0.001
Normal/minimal somatic symptom intensity (Score: 0–4)	107 (7.9)	105 (9.6)	2 (0.8)	
Mild symptoms (Score: 5–9)	367 (27.2)	349 (31.8)	18 (7.1)	
Moderate symptoms (Score: 10–14)	399 (29.6)	339 (30.9)	60 (23.6)	
Severe symptoms (Score: 15–30)	477 (35.3)	303 (27.6)	174 (68.5)	

## Results

### Study population

We included 1671 patients (61.0% of the initial sample), comprising 1609 (96.3%) with a PHQ-9 score ≥ 5 and 62 (3.7%) with a self-reported history of depression taking antidepressants. The sample included 1391 outpatients (83.2%) and 280 inpatients (16.8%). Table 2 shows that outpatients were more often women, older, less educated, more frequently employed or in stable relationships, and had less severe symptoms of depression, anxiety, and somatic complaints. They were also less likely to have a documented history of depression or to experience polypharmacy.

### Use and potentially suboptimal use of antidepressants

Table 3 shows antidepressant use in general was significantly lower among outpatients than inpatients (28.5% vs. 82.5%, p < 0.001). Selective serotonin-reuptake inhibitor (SSRI) use in monotherapy was significantly higher among outpatients (44.1% vs. 25.5%, p < 0.001), whereas combined antidepressant use was significantly higher among inpatients (15.1% vs. 38.5%, p < 0.001). Potentially inadequate treatment response affected a significant proportion of antidepressant users, impacting approximately three-quarters of outpatients (75.1%) and an even higher percentage of inpatients (89.7%). High-risk prescribing was slightly more common in inpatients (25.1%) than outpatients (19.6%), with cardiovascular risks (e.g., QTc prolongation due to citalopram, escitalopram, or TCAs with ≥ 1 additional high-risk drug [12]) being the most frequent ADR in both settings. Fall risk ranked second among outpatients (due to co-medication with drugs like opioids or α-blockers in those aged ≥ 65), while serotonin syndrome risk (from combined serotonergic drugs) was the second most common in inpatients. Potential undertreatment was more prevalent in outpatients (53.3%) than inpatients (20.5%).

**Table 3:** Prevalence of antidepressant use and potentially suboptimal use

	Total	Out-patient setting	In-patient setting	p-value (outpatient vs inpatient)
	No. of patients (%)	No. of patients (%)	No. of patients (%)	
Antidepressant use (missing = 0)	628/1671 (37.6)	397/1391 (28.5)	231/280 (82.5)	< 0.001
SSRI monotherapy	234/628 (37.3)	175/397 (44.1)	59/231 (25.5)	< 0.001
TCA monotherapy	34/628 (5.4)	26/397 (6.5)	8/231 (3.5)	0.099
SNRI/MAOI/Others in monotherapy	211/628 (33.6)	136/397 (34.3)	75/231 (32.5)	0.647
Combined use of ≥ 2 ADs	149/628 (23.7)	60/397 (15.1)	89/231 (38.5)	< 0.001
Potentially inadequate treatment response (missing = 35)	475/593 (80.1)	292/389 (75.1)	183/204 (89.7)	< 0.001
Potential high-risk use (any indicator)	136/628 (21.7)	78/397 (19.6)	58/231 (25.1)	0.109
Potential cardiovascular risk	104/628 (16.6)	58/397 (14.6)	46/231 (19.9)	0.085
Potential risk of serotonin syndrome	29/628 (4.6)	10/397 (2.5)	19/231 (8.2)	0.001
Potential fall risk/orthostatic hypotension	18/628 (2.9)	16/397 (4.0)	2/231 (0.9)	0.022
Potential risk of gastrointestinal bleeding	7/628 (1.1)	7/397 (1.8)	0/231 (0.0)	0.051
Others	5/628 (0.8)	3/397 (0.8)	2/231 (0.9)	1.000
Potential undertreatment	233/554 (42.1)	194/364 (53.3)	39/190 (20.5)	< 0.001

Potentially inadequate treatment response: presence of moderate to severe symptoms of depression (PHQ ≥ 10) despite antidepressant treatment; Other indicators of high-risk use: potential risk of delirium, constipation and hyponatremia; Potential undertreatment: omission of antidepressants in the presence of moderately severe/severe symptoms of depression (PHQ-9 Score ≥ 15)

*SSRI* Selective serotonin-reuptake inhibitors, *TCA* Tricyclic antidepressant, *SNRI* Selective serotonin-norepinephrine reuptake inhibitors, *MAOI* Monoamine oxidase inhibitors, *AD* Antidepressant

### Risk factors for potentially inadequate treatment response

Table 4 shows that among outpatient antidepressant users, higher anxiety levels (adj. OR 1.56; 95% CI 1.36–1.81) and higher somatic symptom severity (adj. OR 1.11; 95% CI 1.00–1.23) were associated with increased risk of potentially inadequate treatment response. Conversely, being in a stable relationship was linked to a reduced risk (adj. OR 0.37; 95% CI 0.17–0.80).

**Table 4:** Risk factors associated with potentially inadequate treatment response

Variables	Odds ratio (95% CI) (crude)	Odds ratio (95% CI) (adjusted)
Outpatient setting		
GAD-7 score	1.69 (1.50–1.90)	1.56 (1.36–1.81)
PHQ-15 score	1.25 (1.17–1.33)	1.11 (1.00–1.23)
Marital status		
Divorced/widowed/single	Reference	Reference
Married or in a stable relationship	0.61 (0.38–0.98)	0.37 (0.17–0.80)

The final model explained 55% of variance in the endpoint (R^2^ = 0.548). Age, gender, current employment status, history of depression, residence, number of drugs and level of general education completed were not significantly associated with potentially inadequate treatment response

### Risk factors for potential high-risk use of antidepressants

Table 5 shows that potential high-risk antidepressant use was associated with higher education levels (adj. OR 2.29; 95% CI 1.20–4.35), more prescribed drugs (adj. OR 1.69; 95% CI 1.46–1.95), TCA monotherapy (adj. OR 3.15; 95% CI 1.06–9.34), and use of two or more antidepressants (adj. OR 3.11; 95% CI 1.54–6.25) rather than SSRI monotherapy. Factors reducing high-risk use included higher PHQ-15 scores (adj. OR 0.92; 95% CI 0.87–0.97), use of SNRI, MAOI, or trazodone rather than SSRI monotherapy (adj. OR 0.11; 95% CI 0.04–0.34), and current employment (adj. OR 0.52; 95% CI 0.29–0.95).

**Table 5:** Risk factors associated with potential high-risk use among patients with antidepressants

Variables	Odds ratio (95% CI) (crude)	Odds ratio (95% CI) (adjusted)
General education		
≤10 y of formal education	Reference	Reference
12–13 y of formal education	1.03 (0.70–1.52)	2.29 (1.20–4.35)
Current employment		
No	Reference	Reference
Yes	0.55 (0.38–0.82)	0.52 (0.29–0.95)
Number of drugs (continuous)	1.50 (1.37–1.64)	1.69 (1.46–1.95)
PHQ-15 Score	1.00 (0.96–1.04)	0.92 (0.87–0.97)
Antidepressant treatment		
SSRI monotherapy	Reference	Reference
TCA monotherapy	4.59 (2.15–9.78)	3.15 (1.06–9.34)
SNRI/MAOI/Others in monotherapy	0.26 (0.12–0.53)	0.11 (0.04–0.34)
Combined use of ≥ 2 ADs	4.82 (3.01–7.74)	3.11 (1.54–6.25)

The final model explained 48% of variance in the endpoint (R^2^ = 0.476). Age, gender, marital status, history of depression, residence, treatment setting, GAD-7 score and PHQ-9 score were not significantly associated with triggering high-risk use

*SSRI* Selective serotonin-reuptake inhibitors, *TCA* Tricyclic antidepressant, *SNRI* Selective serotonin-norepinephrine reuptake inhibitors, *MAOI* Monoamine oxidase inhibitors, *AD* Antidepressant

### Risk factors for potential undertreatment

Table 6 shows that among patients with moderately severe/severe symptoms of depression, potential undertreatment was less likely in those with previous depressive episodes (adj. OR 0.16; 95% CI 0.07–0.38) and those on multiple medications (adj. OR 0.39; 95% CI 0.31–0.49).

**Table 6:** Risk factors associated with potential undertreatment among patients with severe symptoms of depression

Variables	Odds ratio (95% CI) (crude)	Odds ratio (95% CI) (adjusted)
Age (continuous)	1.01 (1.00–1.02)	1.03 (1.00–1.05)
Previous episodes of depression		
No	Reference	Reference
Yes	0.09 (0.05–0.17)	0.16 (0.07–0.38)
Number of drugs (continuous)	0.55 (0.48–0.62)	0.39 (0.31–0.49)

The final model explained 51% of variance in the endpoint (R^2^ = 0.511). Gender, current employment status, marital status, residence, treatment setting, level of general education completed, GAD-7 and PHQ-15 scores were not significantly associated with potential undertreatment

## Discussion

### Summary and interpretation of findings

Our cross-sectional analysis of antidepressant treatment among inpatients and outpatients with symptoms of depression, as included in POKAL subprojects, revealed notable differences in treatment patterns and outcomes. Outpatients were less likely than inpatients to be treated with antidepressants in general, more likely to be treated with SSRIs only, less likely to be treated with a combined antidepressant use, possibly reflecting more severe forms of depression among inpatients.

In our sample, 42% of patients with severe symptoms of depression (median age 42 years; Q1-Q3: 29–55) were potentially undertreated. Underuse affected half of all outpatients, while only one-fifth of inpatients experienced potential undertreatment. Comparatively, a German cohort study reported that 77% of older adults (mean age 69.6 years) with clinical depression were not prescribed antidepressants in 2010 [13]. Similar to our findings, polypharmacy and a self-reported history of depression were protective factors against undertreatment.

Among antidepressant users, potential high-risk use was observed in one fifth of outpatients and one quarter of inpatients, where medication risks, such as drug-drug interactions, may be monitored more closely. High-risk use was independently associated with polypharmacy, use of tricyclic antidepressants, use of two or more antidepressants, and higher levels of education. A Scottish study reported a higher prevalence of high-risk use (34.7%) among 92,601 outpatients in 2019 [14], likely due to its broader set of indicators (28 vs. 12, respectively) and an older study population. However, similar to our findings, cardiovascular events and falls were the most relevant adverse drug reaction risks in both studies, with polypharmacy, TCA use, and multiple antidepressants being key contributors. The higher prevalence of serotonin syndrome risk among inpatients likely reflects their more frequent simultaneous use of multiple antidepressants. Interestingly, our finding that higher education was positively associated with high-risk prescribing, while current employment was protective, is novel but unlikely to represent causal relationships.

Potentially inadequate symptom control on antidepressants affected a significant proportion of antidepressant users and was strongly associated with co-existing symptoms of anxiety and somatic symptoms, which are known risk factors for potential treatment failure [15]. Living in a relationship, however, was found to be protective. The protective effect of being in a relationship aligns with evidence that strong social support and stable life circumstances enhance recovery from depression [16, 17].

### Strengths and limitations

A key strength of this study lies in its comparative analysis of antidepressant use in inpatient and outpatient settings, while simultaneously examining potentially inadequate treatment response, high-risk usage, and undertreatment. However, its external validity is limited due to the non-random sampling of predominantly younger adults. Additionally, data set constraints precluded considering the duration of antidepressant use and drug-disease interactions, which are critical for assessing high-risk usage [12]. Data set limitations also constrained our assessment of treatment response, which would ideally consider antidepressant dosage and treatment history, and clinician-administered depression rating scales, such as the commonly used Hamilton Depression Rating Scale, may be more appropriate for assessing treatment response than the PHQ-9 used here [18, 19]. As such, prevalence estimates should be interpreted with caution. Furthermore, the reliance on pre-specified explicit criteria and limited contextual information identifies potential, rather than actual, inadequate treatment response, undertreatment, and high-risk use. Finally, as with any cross-sectional study, unmeasured confounding may influence the results, and observed associations should not be assumed to be causal.

## Conclusion

Our study underscores the complexities of managing depression in patients with multimorbidity and polypharmacy. The observed undertreatment in severe symptoms of depression in outpatients suggests a need for improved integration of mental health care in these settings. Conversely, the prevalence of high-risk antidepressant use, particularly involving tricyclic antidepressants and multiple concurrent medications, highlights the necessity for cautious prescribing to mitigate adverse drug reactions in both settings. The strong association between potentially inadequate treatment response and co-existing anxiety symptoms emphasizes the importance of comprehensive treatment approaches that address both conditions simultaneously. Our findings highlight a need for personalized, multimodal treatment plans and enhanced clinical vigilance to optimize the use of antidepressants across diverse patient populations. However, more research is desirable to better understand actual opportunities to improve the use of antidepressants in patients with depression and multimorbidity.
